# Constructing a Core Collection of the Medicinal Plant *Angelica biserrata* Using Genetic and Metabolic Data

**DOI:** 10.3389/fpls.2020.600249

**Published:** 2020-12-23

**Authors:** Man Liu, Xin Hu, Xu Wang, Jingjing Zhang, Xubing Peng, Zhigang Hu, Yifei Liu

**Affiliations:** ^1^College of Pharmacy, Hubei University of Chinese Medicine, Wuhan, China; ^2^Hubei Kangnong Seed Co., Ltd., Yichang, China

**Keywords:** *Angelica biserrata*, *Duhuo*, core collection, SSR, coumarin, genetic diversity, germplasm resource

## Abstract

*Angelica biserrata* is an important medicinal plant in Chinese traditional medicine. Its roots, which are known as *Duhuo* in Chinese, are broadly applied to treat inflammation, arthritis, and headache. With increasing market demand, the wild resources of *A. biserrata* have been overexploited, and conservation, assessment of genetic resources and breeding for this species is needed. Here, we sequenced the transcriptome of *A. biserrata* and developed simple sequence repeat (SSR) markers from it to construct a core collection based on 208 samples collected from Changyang-related regions. A total of 132 alleles were obtained for 17 SSR loci used with the polymorphic information content (*PIC*) ranging from 0.44 to 0.83. Abundant genetic diversity was inferred by Shannon’s information index (1.51), observed (0.57) and expected heterozygosity (0.72). The clustering analysis resulted into two sample groups and analysis of molecular variance (AMOVA) showed only 6% genetic variation existed among populations. A further metabolic analysis of these samples revealed the main coumarin contents, such as osthole and columbianadin. According to the genetic and metabolic data, we adopted the least distance stepwise sampling strategy to construct seven preliminary core collections, of which the 20CC collection, which possessed 42 *A. biserrata* individuals accounting for 90.20% of the genetic diversity of the original germplasm, represented the best core collection. This study will contribute to the conservation and management of *A. biserrata* wild germplasm resources and provide a material basis for future selection and breeding of this medicinal plant.

## Introduction

*Angelica biserrata* (R. H. Shan & C. Q. Yuan) C. Q. Yuan & R. H. Shan (Umbelliferae) is a medicinal herb widely used in traditional Chinese medicine (TCM). The medicinal properties of *A. biserrata* are mainly derived from its roots, which are known as *Duhuo* in Chinese and have special aromatic smell when processed. *Duhuo* is broadly applied in TCM to treat inflammation, arthritis, and headache due to its diverse functions of wind-dispelling, dampness-eliminating, cold-dispersing, and pain-relief (Chen et al., 1995; Li et al., 2017; Ma J. H. et al., 2019). Moreover, the aromatic contents of *A. biserrata* are also useful for both medicinal and industrial applications (Zia-Ul-Haq et al., 2014; Senkal et al., 2019). Over 500 compounds have been identified in *A. biserrata*, of which the coumarins osthole and columbianadin, and volatile oils are the main active ingredients (Liu et al., 1995; Yang et al., 2015; Markus et al., 2017; Chen et al., 2018). Osthole and columbianadin are also the index compounds in the 2020 Chinese Pharmacopoeia for quality evaluation of *Duhuo*. These compounds reveal diverse pharmacological effects, including anti-tumor, anti-inflammatory, antioxidant, anti-bacterial, immunomodulatory, sedative, and analgesic effects (Chen et al., 1995; Ma J. H. et al., 2019). Besides its medicinal use, *Duhuo* has been applied in plant protection and cosmetics. Some traditional Chinese wines and teas are made using *Duhuo* as the main raw material (Feng et al., 2018). Alternatively, *Duhuo* is used to produce plant-based pesticides because its ethanol extract has significant inhibitory effects on some bacteria (Feng et al., 2018).

*Angelica biserrata* naturally occurs in south-central and southeast China, with a central distribution area within and around the Wuling Mountains, including the joining region of Hubei, Chongqing, Hunan, and Guizhou Provinces (Zaugg et al., 2011; Guo et al., 2018). Changyang, a county in Hubei Province, is a popular planting area for *A. biserrata*. The Ziqiu town of Changyang is historically known for its role as a local market and circulation terminal for *Duhuo*, which is collected and transported from the surrounding wild and planted areas. Given the good quality and yield of *Duhuo* within and around the Changyang region, “*ZiqiuDuhuo*” has been developed as a Geographical Indication Product for protection. The wild resource of *A. biserrata* within the Wuling Mountains is rich but has been greatly reduced recently due to severe excavation by local people to meet the increasing market demand. Artificial cultivation of *A. biserrata* has been rapidly developed but the medicinal quality of cultivated *Duhuo* is much poorer than that of wild plants. Moreover, as a member of the Umbelliferae plants, the mating system of *A. biserrata* are thought to be outcrossing by wind or insect pollinations (Zych et al., 2019), while self-pollination in field are less successfully (data not shown). Therefore, a comprehensive genetic assessment of the wild and cultivated germplasms is needed for conservation and breeding of this important medicinal herb.

Diverse germplasm resources are critical for crop breeding and improvements (Kumar et al., 2016). However, the conservation and management of genetic resources for most crops are tedious and costly. Establishing core collections provides a convenient solution to conserve and effectively utilize genetic resources (Yuan et al., 2010). The concept of “core collection” was first proposed by Frankel and Brown (1984), and further supplemented and broadened by Brown (1989). A core collection is a subset of germplasm resources that fully represents the genetic diversity of the entire population with the fewest samples (Guzmán et al., 2017; Liu et al., 2019). The core collection should have a sufficiently small sample size, but preserve overall genetic variation. Genetic diversity is the basis of biological diversity which determines the ability of a species to adapt to the external environment and evolve (Ndjiondjop et al., 2017). A lower genetic diversity of a species means less genetic variation and adaptability, which threatens its long-term survival (Linde and Selmes, 2012; Rodríguez-Nevado et al., 2018). Genetic diversity is also an important factor affecting synthesis of secondary metabolites in medicinal plants (Yuan et al., 2010). Therefore, analyzing the genetic diversity of plant populations is a critical step to construct a core collection.

Crops, such as soybean, rice, wheat, peanut, and cucumber (Wang et al., 2011, 2018; Zhang et al., 2011; Kaga et al., 2012; Kobayashi et al., 2016) have been successfully constructed core collections. In contrast, core collections of medicinal plants are still limited although a few have been reported, such as those of *Scutellaria baicalensis* (Bai et al., 2010), *Glycyrrhiza* species (Liu et al., 2020), and *Dalbergia odorifera* (Liu et al., 2019). The results of these studies have consistently revealed the efficiency of core collections for conservation and management of germplasm resources, and to provide material basis for breeding (Campoy et al., 2016; Jeong et al., 2019; Miyatake et al., 2019). Generally, constructing a core collection use both phenotypic trait and molecular marker data. Phenotypic traits are simple and intuitive but unstable and vulnerable to environmental influences, while molecular markers are stable and reliable and more truly reflect genetic differences between germplasms (Xu et al., 2016; Chen et al., 2017; Guo et al., 2017). The metabolic content of medicinal plants is the most important phenotypic trait when constructing a core collection, and therefore is slightly different for that of other traditional crops.

Here, we combined genetic and metabolic analyses of *A. biserrata* samples collected from areas within and around the Changyang region to construct core collection. Due to the limited genomic resources of this medicinal plant, we first conducted RNA-sequencing of a representative sample of *A. biserrata* to develop simple sequence repeat (SSR) markers based on the assembled transcripts to investigate the overall genetic variation of the samples. Moreover, six main kinds of coumarins previously reported in *Duhuo* (Pan et al., 1987; Liu et al., 1995; Yang et al., 2008) were characterized in all corresponding samples used in the genetic analysis. A core collection was finally prepared using data from a least distance stepwise sampling strategy (LDSS) (Wang et al., 2008). Our study will promote the protection and management of the extant *A. biserrata* germplasm resource, and provide core materials for future selection and breeding of this plant. Our method of constructing a core collection will also serve as a reference for other medicinal plants in terms of germplasm conservation and utilization.

## Materials and Methods

### Plant Materials and RNA Extraction

A total of 208 wild *A. biserrata* samples were collected from three different locations of Puling, Gaofeng, and Langping (Supplementary Table 1) in the Changyang region and its adjoining Wufeng country, Hubei Province. These counties have the most concentrated distribution of wild *A. biserrata* in China. Four tissues, including the roots, stems, flowers, and leaves were selected from a representative *A. biserrata* individual, quickly frozen in liquid nitrogen, and stored at −80°C until the RNA-sequencing analysis. Moreover, at least 10 young leaves collected from each individual were dried in silica gel and ground into powder in liquid nitrogen to extract genomic DNA as a template for polymerase chain reaction (PCR) amplification. The roots collected from each plant were sampled and washed with ultrapure water and dried to the no-moisture state in an oven at 50°C. The dried root samples were pulverized into powder with an automatic sample rapid grinding instrument and passed through a 65-mesh sieve to determine the metabolic components.

Total RNA was extracted separately from roots, stems, flowers, and leaves using TRIzol reagent (Thermo Fisher Scientific, Waltham, MA, United States) according to the manufacturer’s instructions and mixed equally to build up the RNA libraries accordingly. The purity, content, and integrity of total RNA were checked with the NanoPhotometer^®^ spectrophotometer (Implen, Westlake Village, CA, United States), a Qubit^®^ Flurometer (Life Technologies, Carlsbad, CA, United States), and the Agilent Bioanalyzer 2100 system (Agilent Technologies, Palo Alto, CA, United States), respectively.

### Transcriptome Sequencing, Assembly and Annotation

For RNA-sequencing, oligo (dT) magnetic beads were used to enrich the eukaryotic mRNA. The first-strand of cDNA was synthesized using random hexamers and dNTPs, DNA polymerase I, and RNase H were then added to synthesize the second-strand, which was further purified using AMPure XP beads. cDNA libraries were prepared according to the manufacturer’s protocol, and sequencing was performed using the Illumina Hiseq 4000 sequencing platform (Illumina, Inc., San Diego, CA, United States). To obtain high-quality RNA-Seq data, the raw sequencing reads were filtered by deleting the invalid reads with adaptor contaminations, reads containing polyA, reads with “N” bases (“N” means that the base information cannot be determined) at a ratio > 5%, and reads with a mass ratio < 5 and a base ratio > 50%.

Trinity software^1^ with default parameters was used to *de novo* assemble the clean data from four plant tissues to generate the contigs and singletons. The functional annotations of the assembled unigenes were performed through BLASTX analyses on the corresponding NCBI non-redundant (Nr) protein^2^ and SWISS-PROT protein^3^ databases with the best similar hit of an E-value of 1e − 05. The classification of protein function was searched in the Gene Ontology (GO) database^4^ and the Clusters of Orthologous Groups (COG) database^5^. The Kyoto Encyclopedia of Genes and Genomes (KEGG) pathway database^6^ was used to determine the biological pathways of unigenes.

### DNA Extraction and SSR Mining

Genomic DNA was extracted by the traditional CTAB method with minor modifications. The quality and purity of the isolated DNA samples were examined by 1% agarose gel electrophoresis and the concentration of each sample was determined using a NanoDrop 2000 spectrophotometer (Ouyang et al., 2018). The final concentration of each DNA sample was diluted to approximately 50 ng/μl for subsequent PCR amplification experiments.

Simple sequence repeats were searched on the assembled unigenes from the transcriptome data using MISA script^7^. The searching standard parameters were set so that mono-, di-, tri-, tetra-, penta-, and hexa-nucleotides were repeated at least 10, 6, 5, 5, 5, and 5 times, respectively. The SSR primers were designed using Primer3 software^8^ with the following parameters (Ma S. et al., 2019): (1) primer length 18–27 bp; (2) PCR product size 100–300 bp; (3) GC content 40–60%; and (4) annealing temperature 55–65°C.

The forward and reverse primers were synthesized commercially, in which the forward primer was added with an M13 tail sequence (GTAAAACGACGGCCAGT) labeling with FAM (blue), HEX (green), and ROX (red). The genomic DNA was PCR amplified in a 10 μl solution whose reaction system contained: 2 μl genomic DNA, 5 μl 2 × *Taq* PCR MasterMix, 0.04 μl forward primer, 0.25 μl reverse primer, 0.15 μl M13-FAM/M13-HEX/M13-ROX, and 2.6 μl ultrapure water. The mixed PCR amplifications were performed in a BiometraTone 96G (Analytik Jena AG, Jena, Germany) with a PCR amplification cycle of 94°C pre-denaturation for 5 min, followed by 35 cycles of 94°C for 30 s, 54°C annealing for 30 s, 72°C extension for 30 s, and a 72°C extension for 10 min. The PCR products were detected by automatic fluorescence using an ABI 3730XL Sequence Analyzer. GeneMapper3.0 software was used to analyze the amplified fragment size of the different samples at each SSR locus.

### High Performance Liquid Chromatography (HPLC) Analysis of the Metabolic Contents

A 0.5 g portion of plant tissue (root) powder was accurately weighed and ultrasound extracted with 20 ml of 50% methanol-dichloromethane solution (v/v) for 30 min at room temperature. Extraction solvent was added, and the extract was shaken and filtered through a 0.45 μm PTFE filter. Standards of umbelliferone (L/N: A04A6L1), 8-methoxypsoralen (L/N: P30J7M16998), bergapten (L/N: W30M9Z57633), columbianetin acetate (L/N: P08F9F54518), osthole (L/N: T08M8B30733), and columbianadin (L/N: P13J9F65515) were purchased from Shanghai Yuanye Bio-Technology Co., Ltd. (Shanghai, China) and were dissolved in 50% methanol-dichloromethane to make stock solutions, which were mixed and diluted with methanol to prepare final standard solutions at a concentration of 100 μg.mL^–1^ for the HPLC analysis.

The chromatographic analysis was performed using an Ultimate XB-C_18_column (250 mm × 4.6 mm, 5 μm; Shanghai Welch Technology Co., Ltd., Shanghai, China) on a Shimadzu LC-20AD HPLC system equipped with SPD-20A detector and CTO-20A thermostatic column compartment (Shimadzu, Kyoto, Japan). The binary gradient elution system consisted of methanol (solvent A) and water (solvent B), and the linear elution procedures were: 0–4 min, 40% A; 4–8 min, 40–50% A; 8–12 min, 50% A; 12–24 min, 50–55% A; 24–28 min, 55% A; 28–32 min, 55–70% A; 32–52 min, 70% A. Column temperature was set to 25°C at a flow rate of 1.0 mL/min, and injection volume was 20 μL. The UV detection wavelength was set to 330 nm. A linear regression analysis was performed with the concentrations of the six coumarin standard solutions as the abscissa (*x*) and the peak area as the ordinate (*y*). The limits of detection (LODs) and limits of quantification (LOQs) were determined at signal-to-noise ratios of 3 and 10, respectively.

### Statistical Analysis

POPGENE version 1.3.2 (Yeh et al., 1997) was used to calculate the genetic diversity parameters, including allele frequency, number of alleles (*Na*), effective number of alleles *(N*e), observed heterozygosity (*Ho*), expected heterozygosity (*He*), Shannon’s information index (*I*), and Nei’ s gene diversity index (*H*). Polymorphism information content (*PIC*) was estimated using PIC Cale software (Nagy et al., 2012). NTSYS-pc software version 2.1 (Rohlf, 2000) was used to perform the cluster analysis using the unweighted pair group method with the arithmetic method (UPGMA) based on similarity matrices calculated according to the simple matching coefficient. GenAlEx version 6.5 (Peakall and Smouse, 2012)was used to perform the principal coordinates analysis (PCoA) based on the Nei’s genetic distance. The population structure was determined using STUCTURE version 2.0 (Pritchard et al., 2000) and further hierarchical analyses of molecular variance (AMOVA) was carried out using GenAlEx.

### Construction of Core Collections

Previous studies have shown that the size of a core collection generally accounts for 5–30% of the original population samples (Brown, 1989; Ortiz et al., 1998; Hu et al., 2000). According to the sample size and the level of genetic diversity of *A. biserrata* in our study, we selected 10–40% of the original population samples as the core sample set. The LDSS method (Wang et al., 2008) was used to identify a group with the smallest genetic distance, and delete the sample with the lowest coumarin content. The seven preliminary core collections with different sampling ratios accounted for 40%, 35%, 30%, 25%, 20%, 15%, and 10% of the original germplasm and were retained by repeated clustering. The retention degree of genetic diversity of these preliminary core collections was manually checked by determining their corresponding genetic diversity indices (*Na*, *Ne*, *Ho*, *He*, *H*, *I*, and *PIC*). The average content of each coumarin and the total coumarins in the seven preliminary core collections were also calculated using GraphPad Prism version 6.0 software (Lazareno, 1994). The core collection was screened for the retention degree of genetic diversity and the coumarin compound contents.

To investigate any significant differences between the core collections and the original samples, SPSS version 24.0 (IBM Crop, 2012) was used to perform a *t*-test on three main genetic parameters (*Ne*, *H*, and *I*) of them. Further comparative analysis of genetic variation between the original germplasm and the core collection or the removed samples (The remainder of the original germplasm after removing the core collection) was also conducted. A PCoA analysis for the original and core germplasms to verify the sample distributions. The difference in coumarin contents between the core collection and original germplasm was assessed by comparing single coumarin and total coumarin contents using GraphPad Prism.

## Results

### Transcriptome Assembly and Annotation of *A. biserrata*

**FIGURE 1:**
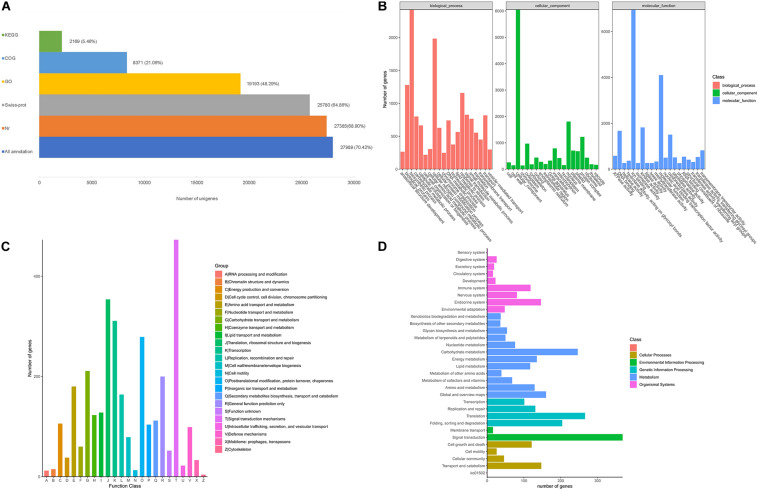
Functional annotation of *A. biserrata* unigenes on the basis of public databases. **(A)** Summary of annotations of unigenes in five databases. **(B)** GO classification of annotated unigenes. **(C)** The COG functional distribution of annotated unigenes. **(D)** Functional classification of unigenes based on the KEGG pathway.

After filtering, a total of 5.9 Gb clean reads were retained and used for the *de novo* assembly, leading to 39,748 unigenes were finally obtained with a N50 sequence length of 1,636 bp, and the percentage of GC was 40.36%. The benchmarking universal single-copy orthologs (BUSCO) analysis based on plant gene models showed that 73.71% of the BUSCO sequences were completely present in the *A. biserrata* transcripts (Supplementary Table 2). About 70.42% (27,989) of the unigenes were annotated in five public databases, in which 27,385 unigenes (68.90%) matched in the Nr database and 25,780 (64.86%) can be annotated in the Swiss-prot database (Figure 1A). Furthermore, a total of 19,193 unigenes (48.29%) could be classified into 57 functional groups categorized in three major GO categories based on sequence homology (Figure 1B). Amongst, biosynthetic process and cellular nitrogen compound metabolic process, nucleus, ion binding are the most abundant GO terms (Figure 1B). We also found that a total of 8,371 unigenes were clustered into 24 function categories in the COG database, and signal transduction mechanisms (473) was the major category, followed by translation, ribosomal structure and biogenesis (354), transcription (311), posttranslational modification, protein turnover, chaperones (279) and Carbohydrate transport and metabolism (211) (Figure 1C). The annotated unigenes of *A. biserrata* were also mapped into 28 KEGG pathways (Figure 1D), of which metabolism was the most represented category. Twelve metabolic pathways were included in the category and the carbohydrate metabolism was the most dominant pathway (Figure 1D).

### Characterization of the Transcriptome Derived SSRs

**FIGURE 2:**
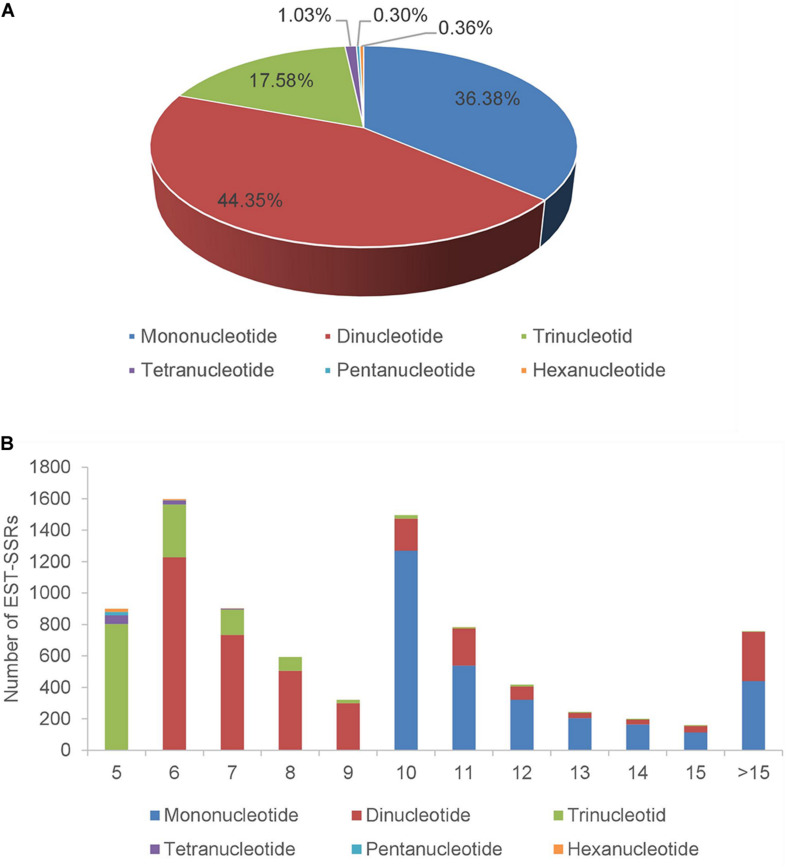
The distribution of SSRs in transcriptome of *A. biserrata*. **(A)** Percentage of different SSRs types in total SSRs. **(B)** The number of SSRs types under different repetition times. *X*-axis represents the times of repetition, and *Y*-axis represents the number of SSRs.

For the assembled *A. biserrata* transcripts, 6,651 sequences contained SSRs and 1,343 sequences contained more than one SSR loci. A total of 8,371 SSR loci were detected with a frequency of 21.06% and an average of one locus per 4.9 kb. Among all SSRs, 7,566 (90.38%) were simple repeat motifs and 805 (9.62%) presented in compound formation (Supplementary Table 3). The repeat types of these SSRs were diverse, of which mononucleotide (3,045, 36.38%) and dinucleotide (3,713, 44.35%) repeats were the major types, followed by trinucleotides (1,472, 17.58%). The total proportion of tetranucleotides (1.03%), pentanucleotides (0.3%), and hexanucleotides (0.36%) was <2% (Figure 2A and Supplementary Table 4). The number of motif repetitions for different SSR types varied broadly, as a whole with a maximum frequency repeat for six repetitions (19.08%), followed by ten (17.86%), seven (10.80%), five (10.74%), and eleven (9.35%) repetitions. Among the three SSR types with the highest frequency, the primary numbers of repetitions of mononucleotides, dinucleotides, and trinucleotides were ten, six and five, respectively (Figure 2B and Supplementary Table 5). Moreover, the A/T repeat type was predominant (96.95%) among mononucleotides, and AG/CT (61.24%) was the primary repeat type among dinucleotides, while AAG/CTT accounted for 23.23% of trinucleotides (Supplementary Table 6).

### SSR Markers Validated for the Genetic Analysis

We randomly selected 146 pairs of SSR primers for primary screening by 1% agarose gel electrophoresis, among which 42 pairs of primers failed to amplify. The remaining primers were further screened using the fluorescent capillary electrophoresis detection method, and 17 pairs of SSR primers with high amplification efficiency, good reproducibility, and high polymorphism were retained for the overall genetic analysis (Supplementary Table 7).

A total of 132 alleles were obtained for all 17 SSR loci among all population samples, and the number of alleles (*Na*) varied from 4 (Abssr107, Abssr128) to 15 (Abssr39) with an average value of 7.76 alleles per locus (Supplementary Table 8). The Abssr39 locus revealed the most abundant genetic variation among the 17 loci investigated, followed by the Abssr33 locus. Both loci had high identification efficiency and therefore are valued for applications to identify *A. biserrata* samples/taxon and closely related species. We further compared the genetic diversity of samples from the three different sampling sites. Although 113 alleles were observed in the Puling population, the overall genetic diversity of the Langping population was the highest among the three sampling sites, suggesting richer genetic variation presented in the *A. biserrata* samples from Langping (Table 1).

**TABLE 1:** Genetic characterization of three wild *A. biserrata* populations.

Location	Sample size	Alleles	*Na*	*Ne*	*Ho*	*He*	*I*	*H*	*PIC*
Puling	70	113	6.65	3.47	0.58	0.67	1.38	0.67	0.64
Gaofeng	69	109	6.41	3.22	0.58	0.65	1.31	0.65	0.61
Langping	69	111	6.53	3.94	0.54	0.73	1.49	0.72	0.69
Total	208	132	7.76	3.89	0.57	0.72	1.52	0.71	0.67

*Na, number of alleles; Ne, effective number of alleles; Ho, observed heterozygosity; He, expected heterozygosity; I, Shannon’s information index; H, Nei’s gene diversity index; PIC, polymorphism information content.*

### Population Structure of the *A. biserrata* Germplasm

**FIGURE 3:**
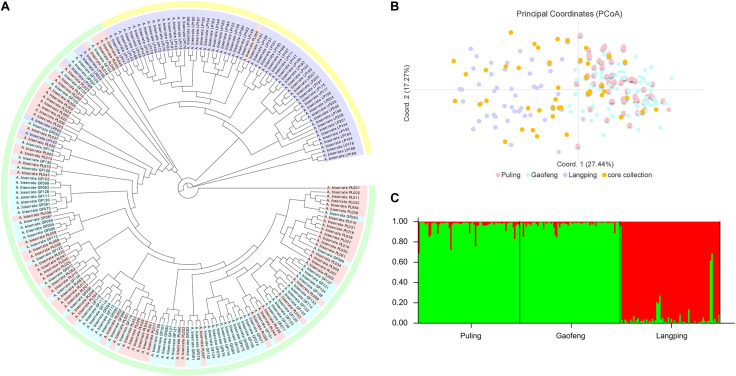
The population structure analysis of 208 *A. biserrata* samples collected from three locations. **(A)** The UPGMA cluster analysis. Light pink, blue and purple colors corresponding to the Puling (PL001-070), Gaofeng (GF071-139), and Langping (LP140-208) populations, respectively. The light green and yellow blocks explain two clustering groups. **(B)** The principal coordinate analysis (PCoA). The light pink, blue and purple dots represent samples collected from the Puling, Gaofeng, and Langping locations, respectively. The orange dot explains the distribution of the core collection materials in the original germplasm. **(C)** The population structure of *A. biserrata* when *K* = 2. Green color explains the first genetic group mainly including samples from the Puling and Gaofeng locations, while red represents the other genetic group with samples from Langping.

The genetic correlation coefficients between the 208 *A. biserrata* samples varied from 0.72 to 0.94, suggesting a similarly genetic background of them. The UPGMA cluster analysis revealed two groups of samples, of which one group included the materials collected from the Puling and Gaofeng locations, while the other group included samples mainly from the Langping (Figure 3A). The PCoA plot showed a similar result (Figure 3B). The first principal coordinate of the PCoA analysis explained 27.44% of the variation, while the second principal coordinate accounted for 17.27% of the variation. The vast majority of the Puling and Gaofeng germplasms were concentrated together in the PCoA plat, while those from Langping were concentrated in another cluster (Figure 3B). In the STRUCTURE analysis, the most appropriate population number for the data set was *K* = 2 (Supplementary Figure 1), suggesting two main genetic groups present for the *A. biserrata* samples investigated. Similarly, the germplasms from the Puling and Gaofeng populations grouped into one genetic group, while those mainly from the Langping population group in the other (Figure 3C). The AMOVA analysis showed that the vast majority of genetic variation was presented within populations, while only 6% of the genetic variation occurred among populations (Supplementary Table 9). The highest level of pairwise *F*_st_ was 0.083, which appeared between the Gaofeng and Langping populations (Supplementary Table 10).

### HPLC Analysis of the Metabolic Contents in Duhuo

The results of the regression equations and the LODs and LOQs for the six coumarin standards are shown in Supplementary Table 11. Precision was evaluated by the relative standard deviation (RSD) values of the peak area of the standard solutions, which were 1.10–1.70%. The RSD repeatability values were 1.39–2.44%, which were indirectly assessed by detecting six samples on the same day. RSD values of stability evaluated in the same sample solution at different time points (0, 2, 4, 6, 8 and 12 h) were <1.89%. A recovery experiment was conducted in which the standards were added to sample powder with known content to evaluate accuracy of the method, and percent recovery was 96.50–102.41%. The peak times of the target peaks on the chromatogram were appropriate with good resolution and there was no negative interference [50% methanol-dichloromethane solution (v/v) as a negative control] (Supplementary Figure 2).

Among the six coumarins investigated in the samples, columbianadin was the most abundant compound (up to 8.27 mg/g in samples), followed by osthole (average 1.17 mg/g), columbianetin acetate (average 0.56 mg/g), 8-methoxypsoralen (average 0.55 mg/g), and bergapten (average 0.20 mg/g). In addition, osthole and columbianadin contents revealed clear differences among *Duhuo* samples (Supplementary Table 12). Among the 208 *Duhuo* samples collected from three sampling locations, most samples from the Puling and Gaofeng population had higher columbianadin content, in particular the Puling049 sample revealed the highest columbianadin content (18.58 mg/g). In contrast, *Duhuo* samples from the Langping location had higher osthole content but relatively lower columbianadin content (Supplementary Table 12).

### Construction of the Core Collection

An LDSS sampling strategy was adopted to obtain seven preliminary core collections named 40CCC, 35CC, 30CC, 25CC, 20CC, 15CC, and 10CC. Each collection included 83, 73, 63, 52, 42, 31, and 21 accessions, accounting for 40%, 35%, 30%, 25%, 20%, 15%, and 10% of the original germplasm, respectively (Table 2). As the sampling ratio decreased, the number of alleles (*Na*) and the retention rates of the alleles (*Ra*) decreased gradually. *Na* ranged from 7.41 (40CC) to 6.65 (10CC) in different collections. Neither 15CC nor 10CC reached 90% of the original germplasm for *Ra*, and others varied from 90.20% (20CC) to 95.50% (40CC) of the original germplasm. The genetic parameters of the seven preliminary core collections were all higher than those estimated for the original germplasm. In particular, the genetic diversity estimates, including all genetic parameters investigated except *Ne*, were higher in 20CC than the others, suggesting better representativeness by the 20CC set than the others (Table 2).

**TABLE 2:** The characteristic in genetic diversity of original germplasm and seven preliminary core collections under different sampling proportions.

Name of sets	Sampling ratio	Size of accessions	*Na*	*Ne*	*Ho*	*He*	*I*	*H*	*PIC*	*Ra* (%)
Original germplasm	100%	208	7.76	3.89	0.57	0.72	1.52	0.71	0.67	100
40CC	40%	83	7.41	4.43	0.58	0.75	1.59	0.75	0.72	95.50
35CC	35%	73	7.24	4.44	0.58	0.75	1.60	0.75	0.72	93.30
30CC	30%	63	7.18	4.49	0.59	0.76	1.61	0.75	0.73	92.53
25CC	25%	52	7.12	4.54	0.60	0.76	1.61	0.75	0.73	91.75
20CC	20%	42	7.00	4.57	0.62	0.78	1.62	0.77	0.74	90.20
15CC	15%	31	6.94	4.58	0.58	0.77	1.62	0.76	0.73	89.43
10CC	10%	21	6.65	4.54	0.55	0.77	1.61	0.75	0.72	85.70

*40–10CC, preliminary core collections obtained with sampling rates of 40% to 10%; Na, number of alleles; Ne, effective number of alleles; Ho, observed heterozygosity; He, expected heterozygosity; I, Shannon’s information index; H, Nei’s gene diversity index; PIC, polymorphism information content; Ra, retention rates of allele.*

**FIGURE 4:**
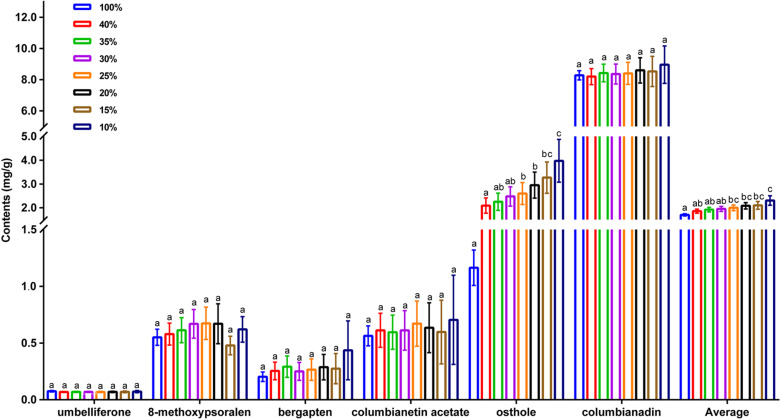
The average content of individual coumarin, average content of total coumarins of the original germplasm and seven preliminary core collections. 100% represents the original germplasm, 40%∼10% represent seven preliminary core collections with different sampling ratios. *X*-axis represents different species of coumarins, *Y*-axis represents content (mg/g).

**FIGURE 5:**
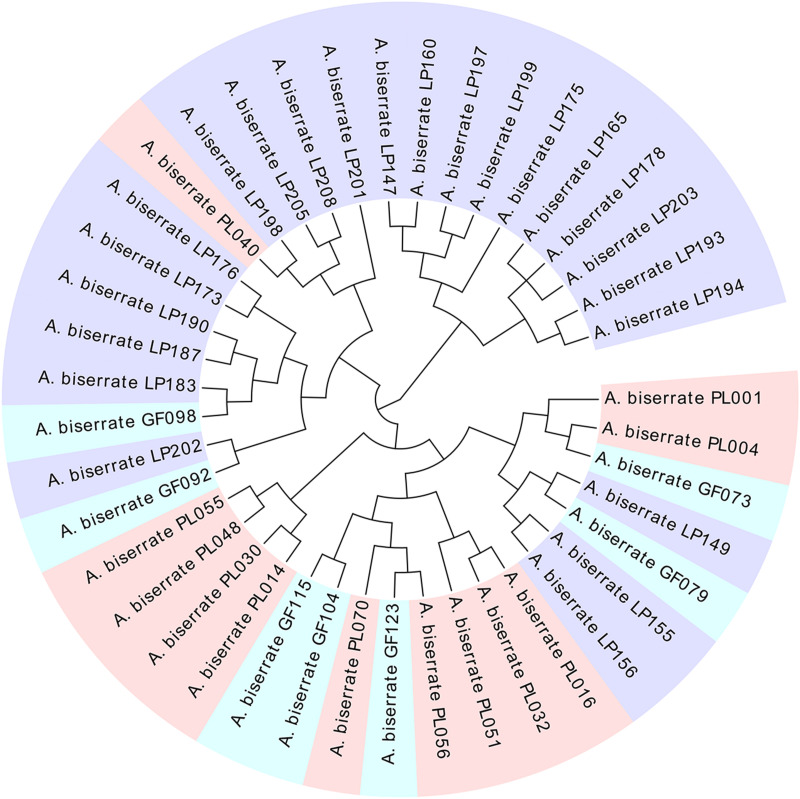
The cluster tree of the core collection (20CC) consisted of 42 germplasms. Light pink, blue, and purple colors represent germplasms collected from Puling, Gaofeng and Langping, respectively.

As the sampling ratio decreased, the average contents of the six coumarins increased gradually (1.81–2.46 mg/g), which may be the reason why individuals with lower total coumarin content were removed during the clustering process. However, when looking at the content of a single coumarin component, umbelliferone content was essentially unchanged, which may have been caused by the low content itself (Figure 4 and Supplementary Table 13). According to the genetic data obtained here, 20CC had the richest genetic diversity among all of the preliminary core collections and retained the maximum genetic diversity of the original germplasm. The average total coumarin contents of 10CC reached the maximum value, followed by 15CC and 20CC. However, the retention rates (*Ra*) of 10CC and 15CC alleles did not reach 90%, which means that the alleles of the original germplasm were not effectively retained, and genetic diversity was not as rich as that of 20CC. In this study, we simultaneously focused on genetic diversity and the metabolic ingredient contents to select the best core collection. Not only the 20CC collection preserved the overall genetic diversity of the original germplasm to the greatest extent, but also the average content of total coumarins increased from 1.81 to 2.20 mg/g compared to the original germplasm. As a consequence, 20CC composed of 42 accessions was the first choice for the best core collection, in which approximately 50% of the germplasm came from the Langping location (Figure 5).

### Quality Evaluation of the Constructed Core Collection

The main genetic parameters, including *Ne*, *H*, and *I* were not significantly different between the core collection and the original germplasm (Supplementary Table 14), indicating no significant genetic difference between them. The retention ratio of the core collection to the original germplasm was greater than 100% for each genetic parameter evaluated, except *Na* (Supplementary Table 15). The seven genetic diversity parameters of the removal samples were all lower than the original germplasm, which means that these removal samples can lead to an increase in the genetic duplication rate of the population (Supplementary Table 15). Moreover, it could be seen from the PCoA diagram that the materials in the core collection were not only similar to the distribution of the original germplasm, but also had a relatively comprehensive and uniform coverage, which represented the geometric distribution of the original germplasm, indicating that the genetic structure of the original germplasm was well maintained by the core collection and further confirming the representativeness of the selected *A. biserrata* core collection (Figure 3B).

## Discussion

### SSR Development of the *A. biserrata* Transcriptome

Microsatellite tandem repeats are widely distributed throughout plant genome and SSR markers derived from these repeats are the most frequently used molecular tool for evaluating plant genetic diversity and population structure (Filippi et al., 2015), for constructing genetic maps (Wu et al., 2014), and for marker-assisted breeding (Kumar et al., 2015). SSRs generally consist of one to six nucleotide basic units repeated multiple times (Feng et al., 2016; Vieira et al., 2016), and are co-dominance with good repeatability and high polymorphism (Li et al., 2014; Ouyang et al., 2018). However, developing SSR markers is relatively difficult in non-model plants without genomic resources (Wang et al., 2019). Most medicinal plants are non-model organisms and their genetic information is scarce, causing a lag in molecular biology and genetic breeding researches (Xin et al., 2017). Given the cost of RNA-seq have fallen dramatically in recent years, transcriptome sequencing therefore provided a convenient and economical way for quickly obtaining SSR markers in non-model plants. Moreover, most SSR markers derived from transcriptome are directly or indirectly related to functional loci. Compared to those SSRs derived from the neutral parts of a plant genome, these transcriptome derived SSRs are potentially more useful, in particular for constructing crop core collections in which selection in relation to focused phenotypic traits is the primary purpose.

Currently, transcriptome derived SSR markers have been widely applied in population genetic analyses of medicinal plants, such as *Picria felterrae* (Yan et al., 2019), *Gynostemma pentaphyllum* (Zhang et al., 2019), and *Perilla frutescens* (Sa et al., 2018). Researches on constructing core collections of medicinal plants using transcriptome derived SSRs are also quickly increased in recent years. An example came from the successful development of a core collection of *Dalbergia odorifera*, in which 19 microsatellite markers developed from its transcriptome were used (Liu et al., 2019). For *A. biserrata*, no SSR markers have been previously reported and the genomic resources of this species is also limited. To our knowledge, this study represented the first report for the genome information (transcriptome) of *A. biserrata*. The transcriptome information and the developed SSR markers presented here therefore provide important molecular resources for future genetic analysis of *A. biserrata* germplasm.

### Population Genetic Variation of *A. biserrata*

Previous studies of *A. biserrata* mainly focused on its chemical and pharmacological aspects, while there are few reports in relation to molecular and genetic diversity of this medicinal plant. Population genetic diversity plays an irreplaceable role in the survival and evolution of organisms and it is extremely important for genetic innovation and breeding of plants. Here, we found a relatively high level of genetic diversity occurred in the wild *A. biserrata* germplasm (average *PIC* value: 0.67, average Shannon’s information index *I*: 1.52; Table 1), which was comparable to those of other plants within the same *Angelica* genus. For example, using a similar method of ten transcriptome derived SSRs, a genetic analysis of 56 *A. dahurica* ecotype samples revealed an average *PIC* value was 0.41 and the Shannon’s information index *I* was 0.85 (Chen et al., 2019). Given the different sample numbers investigated within both analyses, the levels of genetic diversity of these two *Angelica* plants are therefore consistently high.

Our analysis further revealed a relatively higher ratio of genetic variation presented within populations than those between populations (Supplementary Table 10). This result was similar to those reported from *Angelica sinensis* (Zhu et al., 2018) and *Daucus carota* (Chaitra et al., 2020), of which most of genetic variation was found to be present within wild populations rather than between populations. Similar to many other Umbelliferae plants, *A. biserrata* is a cross-pollinated plant, and both the pollen and seeds spread through wind or insects, leading to long-distance dispersal or exchanges of genetic materials between populations is possible. A high level of gene flow therefore possibly occurred across the populations of *A. biserrata* within and around the Changyang region we investigated. Continuously high level of gene flow can strongly weaken population genetic differences and keep a balance of genetic variation between populations in geographically adjacent localities. Further analysis is needed to investigate the population gene flow pattern of this species in future.

### Core Collection Construction of *A. biserrata*

Core collections are the most representative subsamples from the original germplasm, which must be as comprehensive as possible to effectively represent the characteristics of the original germplasm and to ensure that not too much genetic diversity is lost (Frankel and Brown, 1984). For medicinal plants, the intrinsic quality (in terms of the contents of active ingredients) and appearance characteristics are both important phenotypic traits (Huang et al., 2005). Therefore, for constructing a core collection of a medicinal herb, it is necessary to consider both genetic and metabolic diversity simultaneously. Many previous studies of medicinal plants for constructing core collections are only based on genetic diversity, such as those reported in *Scutellaria baicalensis* (Bai et al., 2010) and *Dalbergia odorifera* (Liu et al., 2019). In the present study, we simultaneously determined genetic variation and metabolic contents of the *A. biserrata* germplasm, which may be more comprehensive and indicative for selection of core samples. Moreover, a series of results (Table 2 and Figure 4) further demonstrated that our trade-off method which was mainly based on genetic diversity and supplemented by metabolic components was feasible and reliable.

To construct a core collection of plants, the sampling strategy, including determining a reasonable sampling ratio is also important for the selection of core materials. Different sampling methods can directly affect the extraction of core materials. Due to the good representativeness and stability during the clustering procedure, the LDSS method is currently frequently used as a clustering procedure for constructing core collections of crops. It makes heterogeneous between core materials through deleting maximum genetic duplication of germplasms. Moreover, similar to the maximization (M) strategy which maintains diverse alleles through retaining maximum number of alleles at each locus (Duan et al., 2017), the LDSS method also preserve a sufficient number of alleles on the basis of avoiding genetic duplication to the greatest extent. For medicinal plants such as *A. biserrata*, this method can further combine both genetic and metabolic data in the core collection construction. Moreover, the sampling ratio is often adjusted according to the scale of the original population and richness of genetic diversity during construction of core collections (Hu et al., 2000). Collectively, our strategy of constructing a core collection of *A. biserrata* is valued for selecting materials for conservation and breeding of this plant, and also provides reference for other medicinal plants in terms of core collection construction.

## Data Availability Statement

The datasets presented in this study can be found in online repositories. The names of the repository/repositories and accession number(s) can be found below: https://www.ncbi.nlm.nih.gov/genbank/, PRJNA655389.

## Author Contributions

YL designed the research. XP provided sampling support. XH, XW, and YL contributed to the sampling in the wild. ML and XH performed the experiments. ML analyzed the data and wrote the article. YL, ZH, and JZ revised the manuscript. All authors read and approved the submitted version.

## Conflict of Interest

XP from the Hubei Kangnong Seed Co., Ltd., have done the sampling of most of our samples used for analysis as mentioned in our author contributions. However, his company have not provided any funds for supporting our research. That means, XP is just as a researcher to be involved into our work. The remaining authors declare that the research was conducted in the absence of any commercial or financial relationships that could be construed as a potential conflict of interest.

## Footnotes

1 http://trinityrnaseq.sourceforge.net/

2 ftp://ftp.ncbi.nih.gov/blast/db/FASTA

3 http://www.uniprot.org/

4 http://www.geneontology.org/

5 www.ncbi.nlm.nih.gov/COG

6 https://www.genome.jp/kegg/pathway.html

7 http://pgrc.ipk-gatersleben.de/misa/

8 https://sourceforge.net/projects/primer3

## Supplementary Material

Supplementary Figure 1 STRUCTURE analysis for estimate of the best *K* value. *K* values were set from 1 to 10 and the best one was predicated as *K* = 2 according to the Δ*K* value.

Supplementary Figure 2 Chromatograms of standards, sample and negative sample. **(A)** Mixed solution of each standard. **(B)** *Duhuo* sample. **(C)** 50% methanol-dichloromethane solution (v/v). 1: umbelliferone, 2: 8-methoxypsoralen, 3: bergapten, 4: columbianetin acetate, 5: osthole, 6: columbianadin.

Supplementary Table 1 The specific geographical information of 3 sampling positions of wild *A. biserrata.*

Supplementary Table 2 Transcriptome sequencing data quality and splicing results statistics of *A. biserrata*.

Supplementary Table 3 Distribution of simple sequence repeats (SSRs) in *A. biserrata* transcription.

Supplementary Table 4 Distribution characteristics of different EST-SSR types in transcriptions of *A. biserrata*.

Supplementary Table 5 Distribution characteristics of the different EST-SSRs repeat times observed in the *A. biserrata*.

Supplementary Table 6 Types and Quantities of mononucleotide to trinucleotide EST-SSR repeat motifs in EST-SSR of *A.biserrata*.

Supplementary Table 7 Primer sequence information of 17 pairs of SSR primers.

Supplementary Table 8 Genetic diversity statistics of the 17 microsatellite markers across 208 *A. biserrata* germplasm resources.

Supplementary Table 9 Analysis of molecular variance (AMOVA) for three *A. biserrata* populations.

Supplementary Table 10 The matrix of pairwise genetic differentiation (Fst) among three *A. biserrata* populations.

Supplementary Table 11 Method validation of the HPLC analysis in *Duhuo*.

Supplementary Table 12 The content of osthole and columbianadin among 208 *Duhuo* germplasm resources.

Supplementary Table 13 The average content of each coumarin and total coumarins of different germplasm collections.

Supplementary Table 14 *T*-test results of *Ne, I, H* of original germplasm and core collection.

Supplementary Table 15 Contradistinction of genetic diversity between core collection and removed samples with original germplasm.

## References

[B1] Bai C. K. Wen M. M. Yu F. Li G. S. (2010). Methods on construction of core germplasm collection of *Scutellaria baicalensis* by ISSR marker. *J. Chin. Med. Mater.* 33 1689–1694. 21434427

[B2] Brown A. H. D. (1989). Core collections: a practical approach to genetic resources management. *Genome* 31 818–824. 10.1139/g89-144

[B3] Campoy J. A. Lerigoleur-Balsemin E. Christmann H. Beauvieux R. Girollet N. Quero-García J. (2016). Genetic diversity, linkage disequilibrium, population structure and construction of a core collection of *Prunus avium* L. landraces and bred cultivars. *BMC Plant Biol.* 16:49. 10.1186/s12870-016-0712-9 26912051 PMC4765145

[B4] Chaitra K. C. Sarvamangala C. Manikanta D. S. Chaitra P. A. Fakrudin B. (2020). Insights into genetic diversity and population structure of Indian carrot (*Daucus carota* L.) accessions. *J. Appl. Genet.* 61 303–312. 10.1007/s13353-020-00556-6 32240517

[B5] Chen C. Chen Y. J. Huang W. J. Jiang Y. J. Zhang H. H. Wu W. (2019). Mining of simple sequence repeats (SSRs) loci and development of novel transferability-across EST-SSR markers from de novo transcriptome assembly of *Angelica dahurica*. *PLoS One* 14:e0221040. 10.1371/journal.pone.0221040 31437239 PMC6706007

[B6] Chen D. K. Du Z. Y. Lin Z. R. Su P. Huang H. Y. Ou Z. R. (2018). The chemical compositions of *Angelica pubescens* oil and its prevention of UV-B radiation-induced cutaneous photoaging. *Chem. Biodiv.* 15:e1800235. 10.1002/cbdv.201800235 29996001

[B7] Chen R. K. Hara T. S. Ohsawa R. Yoshioka Y. (2017). Analysis of genetic diversity of rapeseed genetic resources in Japan and core collection construction. *Breed. Sci.* 67 239–247. 10.1270/jsbbs.16192 28744177 PMC5515314

[B8] Chen Y. F. Tsai H. Y. Wu T. S. (1995). Anti-Inflammatory and analgesic activities from roots of *Angelica pubescens*. *Planta Med.* 61 2–8. 10.1055/s-2006-957987 7700984

[B9] Duan H. J. Cao S. Zheng H. Q. Hu D. H. Lin J. Cui B. B. (2017). Genetic characterization of Chinese fir from six provinces in southern China and construction of a core collection. *Scient. Rep.* 7:13814. 10.1038/s41598-017-13219-0 29062029 PMC5653812

[B10] Feng S. G. He R. F. Lu J. J. Jiang M. Y. Shen X. X. Jiang Y. (2016). Development of SSR markers and assessment of genetic diversity in medicinal *Chrysanthemum morifolium* cultivars. *Front. Genet.* 7:133. 10.3389/fgene.2016.00113 27379163 PMC4908101

[B11] Feng Y. M. Li Q. Yang L. Qiu D. Y. (2018). Research and utilization of active ingredients of *Angelicae pubescentis*. *Tradit. Chin. Med.* 7 348–356. 10.12677/TCM.2018.76059

[B12] Filippi C. V. Aguirre N. Rivas J. G. Zubrzycki J. Puebla A. Cordes D. (2015). Population structure and genetic diversity characterization of a sunflower association mapping population using SSR and SNP markers. *BMC Plant Biol.* 15:52. 10.1186/s12870-014-0360-x 25848813 PMC4351844

[B13] Frankel O. H. Brown A. H. D. (1984). “Plant genetic resources today: a critical appraisal,” in *Crop genetic resources: conservation and evaluation*, eds Holden J. H. W. Williams J. T. (London: George Allan and Unwin), 249–257.

[B14] Guo Q. Q. Du G. C. Li Y. X. Liang C. Y. Wang C. Zhang Y. N. (2018). Nematotoxic coumarins from *Angelica pubescens* Maxim. f. *biserrata* Shan et Yuan roots and their physiological effects on *Bursaphelenchus xylophilus*. *J. Nematol.* 50 559–568. 10.21307/jofnem-2018-045 31094158 PMC6909311

[B15] Guo Q. Wang J. X. Su L. Z. Lv W. Sun Y. H. Li Y. (2017). Development and evaluation of a novel set of EST-SSR markers based on transcriptome sequences of black locust (*Robinia pseudoacacia* L.). *Genes* 8:177. 10.3390/genes8070177 28686183 PMC5541310

[B16] Guzmán L. F. Machida-Hirano R. Borrayo E. Cortés-Cruz M. Espíndola-Barquera M. D. Heredia García E. (2017). Genetic structure and selection of a core collection for long term conservation of Avocado in Mexico. *Front. Plant Sci.* 8:243. 10.3389/fpls.2017.00243 28286510 PMC5323459

[B17] Hu J. Zhu J. Xu H. M. (2000). Methods of constructing core collections by stepwise clustering with three sampling strategies based on the genotypic values of crops. *Theoret. Appl. Genet.* 101 264–268. 10.1007/s001220051478

[B18] Huang L. Q. Lv D. M. Yang B. Shao A. J. Chen M. Wei J. H. (2005). Development of the study on germplasm resources of medicinal plants: construction of core collection. *Chin. Mater. Med.* 20 5–26. 16422531

[B19] IBM Crop (2012). *IBM SPSS Statistics for Windows, Version 24.0.* Armonk, NY: IBM Corp.

[B20] Jeong N. Kim K. S. Jeong S. Kim J. Y. Park S. K. Lee J. S. (2019). Korean soybean core collection: Genotypic and phenotypic diversity population structure and genome-wide association study. *PLoS One* 14:e0224074. 10.1371/journal.pone.0224074 31639154 PMC6804985

[B21] Kaga A. Shimizu T. Watanabe S. Tsubokura Y. Katayose Y. Harada K. (2012). Evaluation of soybean germplasm conserved in NIAS genebank and development of mini core collections. *Breed. Sci.* 61 566–592. 10.1270/jsbbs.61.566 23136496 PMC3406788

[B22] Kobayashi F. Tanaka T. Kanamori H. Wu J. Z. Katayose Y. Handa H. (2016). Characterization of a mini core collection of Japanese wheat varieties using single nucleotide polymorphisms generated by genotyping-by-sequencing. *Breed. Sci.* 66 213–225. 10.1270/jsbbs.66.213 27162493 PMC4784999

[B23] Kumar M. Choi J. Y. Kumari N. Pareek A. Kim S. R. (2015). Molecular breeding in Brassica for salt tolerance: importance of microsatellite (SSR) markers for molecular breeding in Brassica. *Front. Plant Sci.* 6:688. 10.3389/fpls.2015.00688 26388887 PMC4559640

[B24] Kumar S. Ambreen H. Variath M. T. Rao A. R. Agarwal M. Kumar A. (2016). Utilization of molecular, phenotypic, and geographical diversity to develop compact composite core collection in the oilseed crop, safflower (*Carthamus tinctorius* L.) through maximization strategy. *Front. Plant Sci.* 7:1554. 10.3389/fpls.2016.01554 27807441 PMC5069285

[B25] Lazareno S. (1994). GraphPad Prism (version 1.02). *Trends Pharmacol. Ences* 15 353–354. 10.1016/0165-6147(94)90038-8

[B26] Li M. Y. Wang F. Jiang Q. Ma J. Xiong A. S. (2014). Identification of SSRs and differentially expressed genes in two cultivars of celery (*Apium graveolens* L.) by deep transcriptome sequencing. *Horticult. Res.* 1:10. 10.1038/hortres.2014.10 26504532 PMC4596314

[B27] Li R. L. Zhao C. Yao M. N. Song Y. Wu Y. Wen A. D. (2017). Analgesic effect of coumarins from Radix *Angelicae pubescentis* is mediated by inflammatory factors and TRPV1 in a spared nerve injury model of neuropathic pain. *J. Ethnopharmacol.* 195 81–88. 10.1016/j.jep.2016.11.046 27915078

[B28] Linde C. C. Selmes H. (2012). Genetic diversity and mating type distribution of *Tuber melanosporum* and their significance to truffle cultivation in artificially planted truffieres in Australia. *Appl. Environ. Microbiol.* 78 6534–6539. 10.1128/AEM.01558-12 22773652 PMC3426713

[B29] Liu F. M. Zhang N. N. Liu X. J. Yang Z. J. Jia H. Y. Xu D. P. (2019). Genetic diversity and population structure analysis of *Dalbergia odorifera* germplasm and development of a core collection using microsatellite markers. *Genes* 10:281. 10.3390/genes10040281 30959931 PMC6523640

[B30] Liu J. H. Xu X. S. Yao X. S. Kobayashi H. (1995). Angelol-type coumarins from *Angelica pubescence* f. *biserrata* and their inhibitory effect on platelet aggregation. *Phytochemistry* 39 1099–1101. 10.1016/0031-9422(95)00045-9 7662273

[B31] Liu Y. L. Geng Y. P. Xie X. D. Zhang P. F. Hou J. L. Wang W. Q. (2020). Core collection construction and evaluation of the genetic structure of Glycyrrhizain China using markers for genomic simple sequence repeats. *Genet. Resour. Crop Evol.* 67 944–941. 10.1007/s10722-020-00944-1

[B32] Ma J. H. Huang J. Hua S. Y. Zhang Y. Zhang Y. W. Li T. T. (2019). The ethnopharmacology, phytochemistry and pharmacology of *Angelica biserrata*-A review. *J. Ethnopharmacol.* 231 152–169. 10.1016/j.jep.2018.10.040 30408534

[B33] Ma S. Dong W. X. Lyu T. Lyu Y. (2019). An RNA sequencing transcriptome analysis and development of EST-SSR markers in Chinese Hawthorn through illumina sequencing. *Forests* 10:82 10.3390/f10020082

[B34] Markus J. Wang D. Kim Y. J. Ahn S. Mathiyalagan R. Wang C. (2017). Biosynthesis, characterization, and bioactivities evaluation of silver and gold nanoparticles mediated by the roots of Chinese herbal *Angelica pubescens* Maxim. *Nanoscale Res. Lett.* 12:46. 10.1186/s11671-017-1833-2 28097599 PMC5241258

[B35] Miyatake K. Shinmura Y. Matsunaga H. Fukuoka H. Saito T. (2019). Construction of a core collection of eggplant (*Solanum melongena* L.) based on genome-wide SNP and SSR genotypes. *Breed. Sci.* 69 498–502. 10.1270/jsbbs.18202 31598083 PMC6776151

[B36] Nagy S. Poczai P. Cernák I. F. Gorji A. M.-H. Géza G. H. Taller J. (2012). PICcalc: an online program to calculate polymorphic information content for molecular genetic studies. *Biochem. Genet.* 50 670–672. 10.1007/s10528-012-9509-1 22573137

[B37] Ndjiondjop M. N. Semagn K. Gouda A. C. Kpeki S. B. Dro Tia D. Sow M. (2017). Genetic variation and population structure of *Oryza glaberrima* and development of a mini-core collection using DArTseq. *Front. Plant Sci.* 8:1748. 10.3389/fpls.2017.01748 29093721 PMC5651524

[B38] Ortiz R. Ruiz-Tapia E. N. Mujica-Sanchez A. (1998). Sampling strategy for a core collection of *Peruvian quinoa* germplasm. *Theoret. Appl. Genet.* 96 475–483. 10.1007/s001220050764 24710887

[B39] Ouyang P. Y. Kang D. L. Mo X. L. Tian E. N. Hu Y. Y. Huang R. S. (2018). Development and characterization of high-throughput EST-based SSR markers for *Pogostemon cablin* using transcriptome sequencing. *Molecules* 23:2014. 10.3390/molecules23082014 30104467 PMC6222658

[B40] Pan J. X. Lam Y. K. Arison B. Smith J. Han G. Q. (1987). Isolation and identification of isoangelol, anpubesol and other coumarins from *Angelica pubescens* Maxim. *Acta Pharmaceu. Sin.* 22 380–384. 3687466

[B41] Peakall R. Smouse P. E. (2012). GenAlEx 6.5: genetic analysis in Excel. Population genetic software for teaching and research-an update. *Bioinformatics* 28 2537–2539. 10.1093/bioinformatics/bts460 22820204 PMC3463245

[B42] Pritchard J. K. Stephens M. Donnelly P. (2000). Inference of population structure using multilocus genotype data. *Genetics* 155 945–959. 10835412 10.1093/genetics/155.2.945 PMC1461096

[B43] Rodríguez-Nevado C. Lam T. T. Holmes E. C. Pagán I. (2018). The impact of host genetic diversity on virus evolution and emergence. *Ecol. Lett.* 21 253–263. 10.1111/ele.12890 29207441

[B44] Rohlf F. J. (2000). *NTSYS-pc, numerical taxonomy and multivariate analysis system, version 2.1.* Setauket, New York: Exeter Software.

[B45] Sa K. J. Choi I. Y. Park K. C. Lee J. K. (2018). Genetic diversity and population structure among accessions of *Perilla frutescens* (L.) Britton in East Asia using new developed microsatellite markers. *Gen. Genom.* 40 1319–1329. 10.1007/s13258-018-0727-8 30105737

[B46] Senkal B. C. Uskutoglu T. Cesur C. Ozavci V. Dogan H. (2019). Determination of essential oil components, mineral matter, and heavy metal content of *Salvia virgata* Jacq. grown in culture conditions. *Turk. J. Agricult. Fores.* 43 395–404. 10.3906/tar-1812-84 31411186

[B47] Vieira M. L. Santini L. Diniz A. L. Munhoz Cde F. (2016). Microsatellite markers: what they mean and why they are so useful. *Genet. Mole. Biol.* 39 312–328. 10.1590/1678-4685-GMB-2016-0027 27561112 PMC5004837

[B48] Wang B. Kumar V. Olson A. Ware D. (2019). Reviving the transcriptome studies: an insight into the emergence of single-molecule transcriptome sequencing. *Front. Genet.* 10:384. 10.3389/fgene.2019.00384 31105749 PMC6498185

[B49] Wang J. C. Hu J. Xu H. M. Zhang S. (2008). A strategy on constructing core collections by least distance stepwise sampling. *Theoret. Appl. Genet.* 115 1–8. 10.1007/s00122-007-0533-1 17404701

[B50] Wang M. L. Sukumaran S. Barkley N. A. Chen Z. B. Chen C. Y. Guo B. Z. (2011). Population structure and marker-trait association analysis of the US peanut (*Arachis hypogaea* L.) mini-core collection. *Theoret. Appl. Genet.* 123 1307–1317. 10.1007/s00122-011-1668-7 21822942

[B51] Wang X. Bao K. Reddy U. K. Bai Y. Hammar S. A. Jiao C. (2018). The USDA cucumber (*Cucumis sativus* L.) collection: genetic diversity, population structure, genome-wide association studies, and core collection development. *Horticult. Res.* 5:64. 10.1038/s41438-018-0080-8 30302260 PMC6165849

[B52] Wu J. Li L. T. Li M. Khan M. A. Li X. G. Chen H. (2014). High-density genetic linkage map construction and identification of fruit-related QTLs in pear using SNP and SSR markers. *J. Exp. Bot.* 65 5771–5781. 10.1093/jxb/eru311 25129128 PMC4203118

[B53] Xin J. Zhang R. C. Wang L. Zhang Y. Q. (2017). Researches on transcriptome sequencing in the study of traditional Chinese medicine. *Evid. Based Compl. Alternat. Med.* 2017:7521363. 10.1155/2017/7521363 28900463 PMC5576426

[B54] Xu C. Q. Gao J. Du Z. F. Li D. K. Wang Z. Li Y. Y. (2016). Identifying the genetic diversity, genetic structure and a core collection of *Ziziphus jujuba* Mill. var. *jujuba* accessions using microsatellite markers. *Scient. Rep.* 6:31503. 10.1038/srep31503 27531220 PMC4987672

[B55] Yan G. Y. Yang F. Bai Y. Y. Li Y. Y. Wu M. L. Almaz B. (2019). Transcriptomic EST-SSR primer development and genetic diversity analysis of *Picria felterrae*. *Chin. Tradit. Herbal Drugs* 50 195–202.

[B56] Yang X. W. Mei G. Q. Wang Y. (2008). Absorption and transport of 6 coumarins isolated from the roots of *Angelica pubescens* f. *biserrata* in human Caco-2 cell monolayer model. *J. Chin. Integr. Med.* 6 392–398. 10.3736/jcim20080413 18405608

[B57] Yang Y. F. Xu W. Song W. Ye M. Yang X. W. (2015). Transport of twelve coumarins from *Angelicae pubescentis* radix across a MDCK-pHaMDR cell monolayer-an in vitro model for blood-brain barrier permeability. *Molecules* 20 11719–11732. 10.3390/molecules200711719 26121397 PMC6332004

[B58] Yeh F. C. Yang R. C. Boyle T. B. J. Ye Z. H. Mao J. X. (1997). *PopGene, the user-friendly shareware for population genetic analysis, molecular biology and biotechnology center.* Canada: University of Alberta.

[B59] Yuan Q. J. Zhang Z. Y. Hu J. Guo L. P. Shao A. J. Huang L. Q. (2010). Impacts of recent cultivation on genetic diversity pattern of a medicinal plant, *Scutellaria baicalensis* (Lamiaceae). *BMC Genet.* 11:29. 10.1186/1471-2156-11-29 20429879 PMC2877650

[B60] Zaugg J. Eickmeier E. Rueda D. C. Hering S. Hamburger M. (2011). HPLC-based activity profiling of *Angelica pubescens* roots for new positive GABAA receptor modulators in Xenopus oocytes. *Fitoterapia* 82 434–440. 10.1016/j.fitote.2010.12.001 21147202

[B61] Zhang H. L. Zhang D. L. Wang M. X. Sun J. L. Qi Y. W. Li J. J. (2011). A core collection and mini core collection of *Oryza sativa* L. in China. *Theoret. Appl. Genet.* 122 49–61. 10.1007/s00122-010-1421-7 20717799

[B62] Zhang X. Su H. L. Yang J. Feng L. Li Z. H. Zhao G. F. (2019). Population genetic structure, migration, and polyploidy origin of a medicinal species *Gynostemma pentaphyllum* (Cucurbitaceae). *Ecol. Evolut.* 9 11145–11170. 10.1002/ece3.5618 31641462 PMC6802062

[B63] Zhu T. T. Jin L. Huang D. D. Lu Y. Y. Li J. T. Sun S. B. (2018). Comparative study on populations genetic diversity between wild and cultivated A*ngelica sinensis*. *Chin. Tradit. Herbal Drugs* 49 211–218.

[B64] Zia-Ul-Haq M. Ahmad S. Bukhari S. A. Amarowicz R. Ercisli S. Jaafar H. Z. E. (2014). Compositional studies and biological activities of some mash bean (*Vigna mungo* (L.) Hepper) cultivars commonly consumed in Pakistan. *Biol. Res.* 47:23. 10.1186/0717-6287-47-23 25028256 PMC4101733

[B65] Zych M. Junker R. R. Nepi M. Stpiczynska M. Stolarska B. Roguz K. (2019). Spatiotemporal variation in the pollination systems of a supergeneralist plant: is *Angelica sylvestris* (Apiaceae) locally adapted to its most effective pollinators? *Anna. Bot.* 123 415–428. 10.1093/aob/mcy140 30059963 PMC6344219

