# Supplementary figures and images for: Constructing a Core Collection of the Medicinal Plant Angelica biserrata Using Genetic and Metabolic Data
Source: Front Plant Sci. 2020 Dec 23;11:600249. doi: 10.3389/fpls.2020.600249 (PMC7785966; doi:10.3389/fpls.2020.600249)

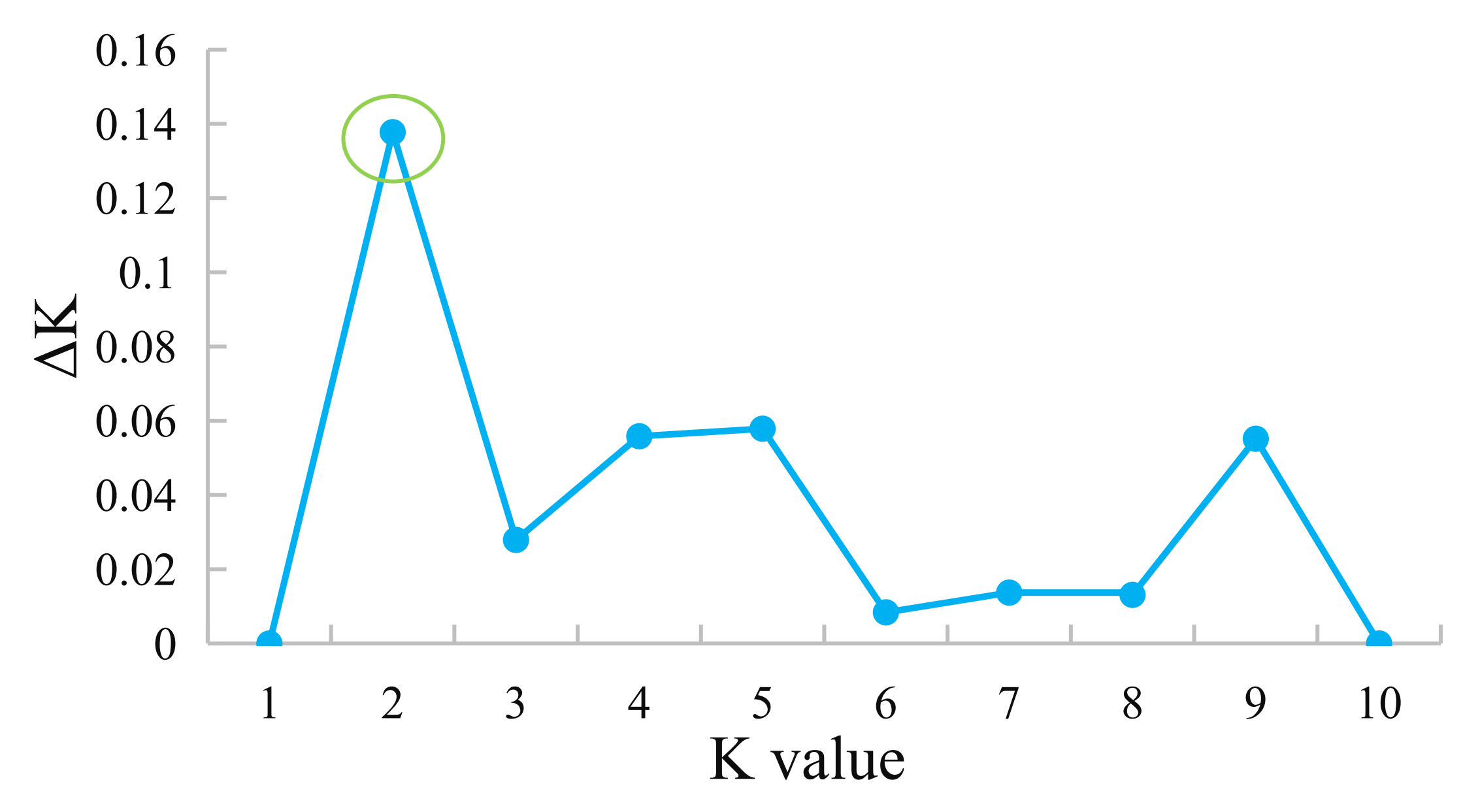

Supplement: Supplementary Figure 1 — STRUCTURE analysis for estimate of the best K value. K values were set from 1 to 10 and the best one was predicated as K = 2 according to the ΔK value. [file Image_1.TIF]

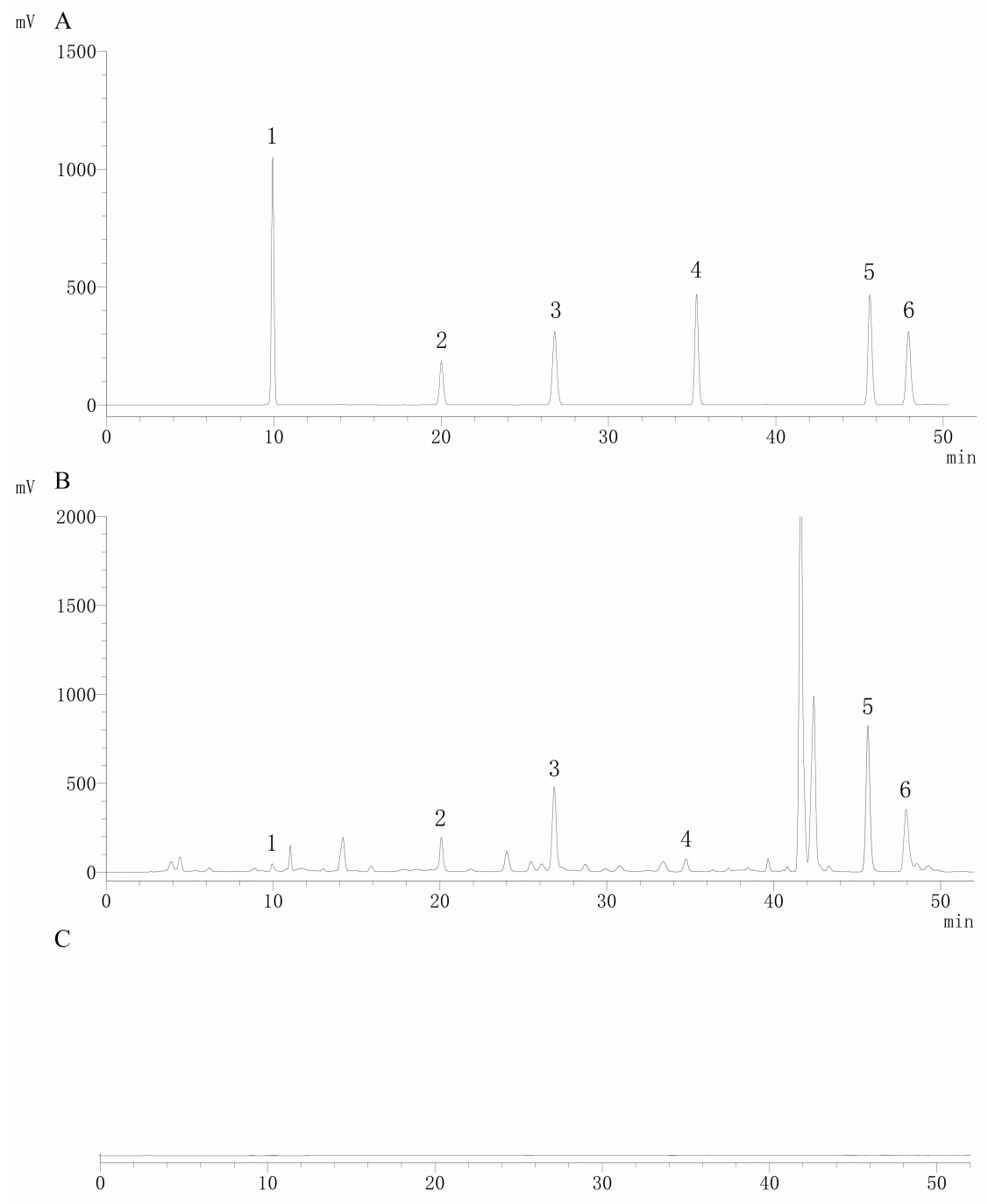

Supplement: Supplementary Figure 2 — Chromatograms of standards, sample and negative sample. (A) Mixed solution of each standard. (B) Duhuo sample. (C) 50% methanol-dichloromethane solution (v/v). 1: umbelliferone, 2: 8-methoxypsoralen, 3: bergapten, 4: columbianetin acetate, 5: osthole, 6: columbianadin. [file Image_2.TIF]
